# Advancing *Pistacia terebinthus* L. (*Anacardiaceae*) Research: Food Preservation, Functional Foods, and Nutraceutical Potential

**DOI:** 10.3390/foods14071245

**Published:** 2025-04-02

**Authors:** Daniela Batovska

**Affiliations:** Institute of Chemical Engineering, Bulgarian Academy of Sciences, Acad. G. Bonchev Str., Bl. 9, 1113 Sofia, Bulgaria; danielabatovska@gmail.com

**Keywords:** antioxidant, antimicrobial, bioactive compounds, food technologies, turpentine tree

## Abstract

*Pistacia terebinthus* L., commonly known as the turpentine tree, is a wild-growing species with a well-documented history of use in traditional medicine and ethnobotany. Various parts of the plant—fruits, seeds, resin, leaves, and galls—have demonstrated significant bioactive potential, particularly antioxidant, antimicrobial, and functional properties. Despite these promising attributes, the industrial application of *P. terebinthus* L. in contemporary food and nutraceutical systems remains limited and underexplored. Recent advances have employed a range of technological strategies—including encapsulation, active food packaging, emulsion stabilization, probiotic immobilization, and fermentation—to improve the stability, bioavailability, and functional performance of *P. terebinthus* L.-derived extracts within food matrices. These approaches have shown potential in enhancing aroma retention, extending shelf life, and supporting probiotic viability, thereby positioning *P. terebinthus* L. as a promising candidate for use in functional formulations and natural food preservation. Nevertheless, further investigation is required to optimize processing parameters, assess the long-term stability of bioactive compounds, and establish standardized regulatory frameworks. Addressing these challenges will be essential for facilitating the broader integration of *P. terebinthus* L. into the functional food, nutraceutical, and food preservation industries.

## 1. Introduction

*Pistacia terebinthus* L., commonly referred to as the terebinth or turpentine tree, is a wild-growing deciduous shrub native to Mediterranean ecosystems [1]. Known for its distinctive aroma, bioactive richness, and historical importance, various parts of the tree—including fruits, seeds, resin, leaves, and galls—have long been used in traditional food, medicine, and natural preservation practices [2,3,4,5,6,7].

Despite its rich ethnobotanical heritage and demonstrated functional properties, *P. terebinthus* L. remains underrepresented in modern food research and industrial applications. Previous reviews have primarily focused on its traditional use, general phytochemical composition, and pharmacological activities [2,5,8,9], with limited attention to its integration into emerging food technologies or sustainable formulations. A recent review highlighted several *Pistacia* species, including *P. terebinthus* L., in the context of leaf valorization for natural food preservation [7]; however, a focused evaluation of this species—particularly regarding the broader food and nutraceutical applications of its diverse plant parts—remains lacking.

This review addresses that gap by providing a focused synthesis of recent developments concerning *P. terebinthus* L., especially in relation to its potential for flavor enhancement, food preservation, functional food development, and sustainability. Special emphasis is placed on technological innovations—such as encapsulation, probiotic immobilization, fermentation, and bioactive stabilization—that have improved the bioavailability, stability, and application of *P. terebinthus* L.-derived extracts in food systems.

**Figure 1:**
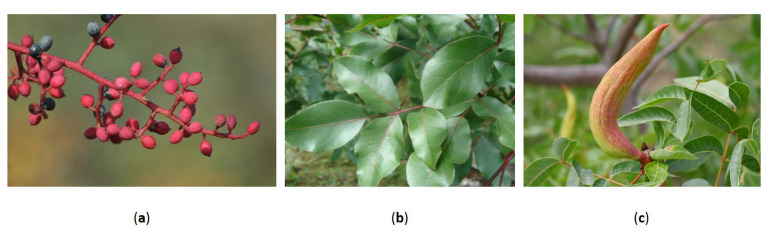
Representative plant parts of *P. terebinthus* L. From left to right: (**a**) Infructescence with mature red and black drupes (photo by Zeynel Cebeci, Ayaş, Erdemli–Mersin, Turkey; Wikimedia Commons, CC BY-SA 4.0); (**b**) Glossy pinnate leaves (photo by Fernando Losada Rodríguez, Monfragüe National Park, Spain; Wikimedia Commons, CC BY-SA 4.0); (**c**) aphid-induced gall (likely *Baizongia pistaciae*) on the leaf rachis (photo by © Entomart, used with permission). These plant parts are closely associated with the species’ traditional uses and phytochemical richness.

One of the most distinctive components of the turpentine tree is its small, resinous drupes (Figure 1a), which encloses a nutrient-rich seed. These fruits have long featured in traditional Mediterranean diets and are still widely consumed in Turkey, where they are eaten fresh as an appetizers or incorporated into dishes such as *bulgur pilaf*, *pastries*, and *balbeki* [4]. Roasted fruits are used to make *menengiç coffee*, a traditional caffeine-free beverage known for its velvety texture and nutty flavor [10]. Other culinary applications include pickling the unripe fruits or drying and grinding them into a mildly resinous spice used in meat and seafood dishes [4,11].

The seeds enclosed within the fruit are highly valued for their nutty, aromatic profile, making them a notable source of oil extraction. Cold-pressed terebinth oil, commonly utilized in Mediterranean cuisine, is prized for its distinctive flavor and is frequently incorporated into salads, cheeses, and marinades [12]. Ground seeds are also incorporated into pastries and confections, particularly in Turkish and Greek culinary traditions [13].

Beyond its fruits and seeds, the resin of *P. terebinthus* L. has historical value in both culinary and medicinal contexts. Traditionally, the resin is obtained by incising the bark, and allowing the exudate to harden upon exposure to air before collection [3,13]. The resin has been widely utilized as chewing gum, a flavoring agent in fermented beverages and confections, and as a substitute for Chios mastic gum, which is derived from *P. lentiscus* L. [3,14,15]. In traditional medicine, *P. terebinthus* L. resin has been employed for lip care, gastrointestinal relief, and respiratory infections [14].

The leaves of *P. terebinthus* L. (Figure 1b), although less prominent in culinary use, have traditionally been prepared as herbal infusions for their digestive benefits or used as natural food wraps, similar to vine leaves, in slow-cooked dishes [4,6]. Insect-induced galls formed on the leaves (Figure 1c) have garnered scientific interest due to their potent antimicrobial activity, with potential applications in infection control [16]. Traditionally, these galls have been used as diuretics, astringents, and stimulants in the management of asthma and urinary tract infections [17].

Within the genus *Pistacia*, *P. vera* L. is widely cultivated for its edible seeds, while *P. lentiscus* L. is commercially exploited for its triterpenoid-rich mastic gum [18,19]. Wild relatives such as *P. atlantica* Desf. have also attracted growing interest for their seed oil and oleoresins, with recent studies detailing the phenolic and lipophilic composition of their roots, buds, and fruits, along with associated antimicrobial, antioxidant, anti-inflammatory, and antityrosinase activities [20,21,22]. In contrast, *P. terebinthus* L. remains largely uncultivated and underexplored, despite its long-standing traditional use and notable phytochemical diversity. Documented bioactive constituents of *P. terebinthus* L. include flavonoids, phenolic acids, fatty acids, and terpenoids, distributed across multiple plant parts such as fruits, resin, leaves, and galls. This multi-organ presence of functionally relevant compounds highlights its potential as a versatile and underutilized source of natural ingredients for food preservation and functional formulation development.

Building on this potential, *P. terebinthus* L. constitutes a versatile natural source of bioactives with diverse health-promoting properties. Its complex biochemical profile positions it as a strong candidate for innovation in food and nutraceutical systems, particularly in response to the growing demand for clean-label, plant-based functional ingredients [5].

This review aims to (i) synthesize current scientific knowledge on *P. terebinthus* L., particularly in relation to its application in food and nutraceutical systems; (ii) highlight technological advancements that improve the functionality and bioavailability of its bioactive compounds; and (iii) identify existing research gaps and propose future directions for industrial and scientific exploration. In doing so, the review offers a targeted, application-oriented perspective that distinguishes it from previous general overviews of the species.

A structured literature search was conducted using PubMed, Scopus, Web of Science, and Google Scholar to identify peer-reviewed research articles, reviews, and books related to the terebinth. Search terms included “*Pistacia terebinthus* L.”, “bioactive compounds”, “functional foods”, “food preservation”, “nutraceuticals”, and “antimicrobial activity”. Priority was given to publications from the past 10 years, with older sources included where relevant for historical and contextual background. A total of 51 articles were selected based on their relevance, scientific rigor, and contribution to the field.

Notably, the modest number of recent studies underscores the limited scientific attention that *P. terebinthus* L. has received to date, highlighting the need for further research on this underexplored species.

## 2. Bioactive Profile and Nutritional Potential of *P. terebinthus* L.

The turpentine tree harbors a diverse range of bioactive compounds, contributing to its antioxidant, antimicrobial, and overall health-promoting properties. The composition and concentration of these bioactive constituents vary across different plant parts, each offering distinct functional and nutraceutical potential. Table 1 provides a brief summary of these bioactive compounds and their associated properties. The following sections detail the key characteristics and functional relevance of each plant part.

### 2.1. Fruits and Seeds

The fruits of *P. terebinthus* L. contain a complex array of bioactive compounds, with both the outer layer (pericarp) and the seed contributing to their nutritional value and functional properties. These components are rich in essential fatty acids, phenolic compounds, flavonoids, and terpenoids, which are known for their antioxidant, antimicrobial, and anti-inflammatory activities.

Cold-pressed oil extracted from Turkish *P. terebinthus* L. fruits is particularly nutrient-rich, containing protein (10%), cellulose (18%), and fiber (11%), making it a valuable dietary supplement in Japanese quail nutrition [4,23]. Additionally, the oil exhibits natural antioxidant properties, attributed to its high concentrations of vitamin C and γ-tocopherol [5].

Beyond its antioxidant potential, *P. terebinthus* L. oil serves as a valuable source of essential minerals, including potassium, calcium, sodium, and phosphorus, which play crucial roles in bone health, electrolyte balance, oxygen transport, and metabolic functions [5,24].

As *P. terebinthus* L. fruits mature from green to black, their fatty oil content increases from 13% to 37%, with a concurrent rise in oleic and palmitic acid levels, while linolenic acid levels decline. Meanwhile, essential oil content decreases from 0.12% to 0.08%, accompanied by compositional shifts in dominant volatile compounds. At the green fruit stage, the primary constituents include *p*-cymen-8-ol and *p*-anisaldehyde. As the fruit ripens to a red–black stage, limonene becomes the predominant compound. By the black fruit stage, α-pinene emerges as the dominant volatile, marking its peak aromatic potential. These findings suggest that harvesting at full ripeness optimizes oil quality and aromatic composition. To maintain consistency and maximize yield, proper harvester training is recommended [27].

The composition of essential oil in *P. terebinthus* L. fruits exhibits geographical variation, influencing its bioactivity and potential applications. The oil extracted from Kosovar fruits is particularly rich in (*Z*)-β-ocimene (45%), limonene (24%), and α-pinene (13%). This oil demonstrates four times greater antibacterial activity against methicillin-sensitive *Staphylococcus aureus* (MIC_50_ = 0.016% *v*/*v*) compared to tea tree oil, reinforcing its traditional use as an anti-inflammatory and antiseptic agent [26].

The *n*-hexane extract from Turkish fruits is characterized by a high concentration of oleic (53%), palmitic (22%), and linoleic (19%) acids. This extract has demonstrated acetyl- and butyryl cholinesterase inhibitory activity, suggesting potential neuroprotective effects. Additionally, the alcohol-aqueous extract exhibited stronger cholinesterase inhibition than galanthamine, along with significant antioxidant activity in cupric reducing antioxidant capacity (CUPRAC) assays, indicating potential benefits for cognitive and cardiovascular health [25]. The acetone extract derived from the fruit shell demonstrated significant inhibitory activity against α-glucosidase and α-amylase, with IC_50_ values of 0.19 mg/mL and 23.49 mg/mL, respectively. Luteolin was identified as the primary bioactive compound responsible for this effect [28].

The ethanol extract of *P. terebinthus* L. fruits has shown potent antimicrobial and antifungal properties, making it a promising natural additive for various industries. It demonstrated strong antibacterial activity against *Listeria monocytogenes* (22.39 mm inhibition zone) and *Vibrio anguillarum* A4 (17.67 mm), surpassing conventional antibiotics such as amikacin and gentamicin. The extract also eliminated *Candida glabrata* at 20 mg/mL within 24 h, supporting its potential applications in the food, pharmaceutical, and cosmetic industries [35].

The methanol extract of *P. terebinthus* L. fruits exhibited strong antioxidant activity, with 71% inhibition in both 2,2-diphenyl-1-picrylhydrazyl (DPPH) and 2,2′-azino-bis(3-ethylbenzothiazoline-6-sulfonic acid (ABTS) assays and a ferric-reducing antioxidant power (FRAP) value of 18 mg FeSO_4_ eq/g. Additionally, it demonstrated α-glucosidase inhibition (IC_50_ = 2.14 mg/mL) and exhibited antibacterial activity against *S. aureus* and *Escherichia coli* in disk diffusion tests. These bioactivities are attributed to its high vitamin C content and key phenolic compounds, including rutin, gallic acid, and syringic acid, underscoring its antioxidant, flavor-enhancing, and food-preserving properties [5]. Furthermore, sonication-assisted extraction enhanced the yield of quercetin and catechol, further contributing to the extract’s antioxidant potential [24].

Crude oil extracted from *P. terebinthus* L. seeds using the Soxhlet method (47%) contains oleic (46%), linoleic (24%), and palmitic (24%) acids, with a composition comparable to the *n*-hexane fruit extract [29,30]. Additionally, cold-pressed oil, obtained through optimized extraction conditions, yielded protein (18.1%), fat (5.4%), moisture (7.7%), and ash (4.4%). The optimal conditions for high-purity protein isolate extraction were pH 8, 50 °C, and a 60 min extraction period. These protein isolates exhibited excellent oil absorption, foaming, and emulsifying properties, along with high thermal stability, making them suitable for use in muffins and other food applications [12].

The fruits and seeds of *P. terebinthus* L. represent a rich and versatile source of bioactive compounds, including essential fatty acids, phenolic antioxidants, and functional proteins. The oils and extracts demonstrate a broad spectrum of health-promoting properties, such as antimicrobial, neuroprotective, cardioprotective, and antidiabetic activities. The high nutritional value, combined with proven efficacy in enzyme inhibition and antioxidant assays, highlights their potential in functional foods, dietary supplements, and natural preservatives. Moreover, the unique compositional shifts during fruit ripening and the successful enhancement of bioactives through extraction techniques (e.g., sonication) point to promising strategies for valorizing these underutilized plant parts in food, cosmetic, and nutraceutical industries.

### 2.2. Resin

Although *P. terebinthus* L. resin has been traditionally utilized for its aromatic and medicinal properties, research on its bioactive potential remains relatively limited. However, recent studies indicate that the resin possesses notable antimicrobial and preservative properties, suggesting its potential applications in food preservation and safety.

Beyond essential oils such as limonene and α-pinene, the resin contains a rich profile of triterpenoids, including isomasticadienonic acid, 28-norolean-17-en-3-one, and masticadienonic acid [31]. These compounds exhibit a broad spectrum of functional properties—anti-inflammatory, antimicrobial, antioxidant, antitumor, and gastroprotective—which highlight the resin’s relevance in therapeutic and food preservation contexts. Further studies are warranted to explore the mechanisms and stability of these compounds in various formulations.

### 2.3. Leaves

Essential oil extracted from *P. terebinthus* L. leaves exhibits significant antimicrobial activity, with composition varying by geographical origin. Kosovar leaf oil is particularly rich in α-pinene (33%), limonene (29%), and (*Z*)-β-ocimene (29%) and has shown twice the antibacterial activity of tea tree oil (MIC_50_ = 0.03% *v*/*v*), highlighting its potential as a natural food preservative [26]. Interestingly, the essential oil from Tunisian leaves shares a nearly identical composition, suggesting a degree of phytochemical consistency across certain populations. In contrast, Sardinian oil displays a slightly different profile, characterized by α-pinene (35%), camphene (2%), β-pinene (5%), terpinolene (35%), and β-phellandrene (5%). Both Tunisian and Sardinian oils have demonstrated fungicidal activity against *Candida* spp., reinforcing the antimicrobial potential of *P. terebinthus* L. essential oils in food preservation [32].

Beyond volatile oils, *P. terebinthus* L. leaf extracts demonstrate a wide spectrum of bioactivities with implications for human health. Extracts rich in flavonoids such as luteolin and rutin have shown inhibitory effects on pancreatic triacylglycerol lipase (IC_50_ = 35 µg/mL) and starch-digesting enzymes (IC_50_ = 9 µg/mL), along with cytotoxicity against colorectal cancer cell lines HT29 (IC_50_ = 99 µg/mL) and SW480 (IC_50_ = 56 µg/mL), suggesting their relevance in metabolic and cancer-related applications [33].

The phytochemical profile of the leaves is particularly diverse, including triterpenoids such as masticadienediol, masticadienolic acid, masticadienonic acid, 3-epimasticadienolic acid, tirucallone, oleanolic acid, and morolic acid—compounds known for their antioxidant and anti-inflammatory effects [8]. Leaves also contain substantial amounts of total phenols (29–65 mg GAE/kg dry matter), flavonoids (11–18 mg CE/kg), and hydrolysable tannins (12–39 mg TAE/kg), all contributing to their antioxidant potential. Soxhlet extraction with solvents of decreasing polarity—water, ethanol, ethyl acetate, and *n*-hexane—revealed a decline in extract yield, reflecting the prevalence of polar compounds in the leaf matrix. Importantly, the leaf material contains approximately double the levels of phenolics, flavonoids, and tannins compared to fruits, underscoring its superior suitability for functional applications [36].

Recent work has expanded understanding of this plant part’s bioactivity. Methanolic and ethyl acetate maceration, along with aqueous decoction followed by lyophilization, yielded extracts rich in bioactive compounds [34]. Total phenolic content ranged from 13 to 42 mg GAE/kg dry matter, and flavonoids from 11 to 34 mg RE/kg, with the highest values in methanolic and ethyl acetate extracts, respectively. Antioxidant tests confirmed the potency of methanol extracts (ABTS: 3.29 mmol TE/g; DPPH: 2.06 mmol TE/g; CUPRAC: 2.67 mmol TE/g; FRAP: 1.62 mmol TE/g) along with high metal-chelating activity (65.65 mg EDTA/g). These extracts also inhibited key metabolic and neurological enzymes, including acetylcholinesterase (AChE), butyrylcholinesterase (BChE), α-amylase, α-glucosidase, and tyrosinase, positioning them as promising agents for neuroprotective, metabolic, and cosmetic applications. A total of 35 bioactive compounds have been identified in the leaves, including phenolic acids (gallic, digallic, protocatechuic, and *p*-coumaric acids), procyanidin B, and various flavonoids such as taxifolin, myricetin, quercitrin, quercetin, cosmosiin, and luteolin. Of particular note, rutin and luteolin have been directly linked to the strong antioxidant capacity of the leaf extracts and their potential roles in food preservation and glucose metabolism regulation [33].

Taken together, the essential oils and extracts from *P. terebinthus* L. leaves present a multifaceted bioactive profile, combining potent antioxidant, antimicrobial, enzyme-inhibitory, and anti-inflammatory properties. These attributes highlight the high potential of the leaves as ingredients in functional foods, natural preservatives, and plant-based health formulations.

While major monoterpenes such as α-pinene, β-pinene, and limonene, that are abundant in the essential oils, are classified as generally recognized as safe (GRAS) and approved for use as flavoring agents by regulatory authorities such as the U.S. Food and Drug Administration (FDA) [37,38], some of their oxidized derivatives, particularly those derived from limonene, have been reported as sensitizers in topical applications, including cosmetics and pharmaceuticals [39,40]. Although adverse effects via oral intake are uncommon in healthy individuals, the potential for allergenic reactions in sensitive populations or under oxidative stress conditions warrants consideration. This underscores the need to assess the oxidative stability of terpene-rich essential oils and their interactions with food matrices to ensure safe and effective use in food systems.

In contrast, other constituents of *P. terebinthus* L., particularly triterpenoids such as oleanolic acid, ursolic acid, and lupeol, have shown promise in alleviating food allergy responses. These compounds exert their beneficial effects through a range of mechanisms, including modulation of immune signaling during sensitization, strengthening of epithelial barrier function, inhibition of effector cell activation, attenuation of oxidative stress, and regulation of gut microbiota [41]. These findings position triterpenoid-rich plant extracts as valuable components in the development of functional foods targeting food allergy management and immune health.

### 2.4. Galls

*Pistacia terebinthus* L. galls are formed through aphid-induced manipulation, where specific aphid species stimulate the tree to produce monoterpenes and other bioactive compounds that contribute to their own protection and development. These galls have been identified as a rich source of bioactive constituents, exhibiting potential applications in food preservation, agriculture, and pharmaceuticals [17].

Essential oil extracted from Kosovar *P. terebinthus* L. galls is particularly rich in α-pinene (66%), limonene (17%), and β-pinene (11%), and has demonstrated strong antibacterial activity against *S. aureus* (MIC_50_ = 0.032% *v*/*v*). These findings suggest promising applications for natural food preservation [26].

Additionally, the methanol extract of Algerian galls has exhibited growth-inhibitory effects on *Allium cepa* L. root development, an activity attributed to the presence of caffeoylquinic acid, further emphasizing the bioactive potential of these structures [17]. Furthermore, the extract (50 µL) displayed bactericidal activity against *S. aureus*, producing inhibition zones of 20 mm [42].

Similarly, ethanol extracts of Turkish galls have shown growth inhibition of *S. aureus* and *E. coli*. The observed bioactivity of these extracts is likely attributed to the presence of lauric, myristic, and arachidonic acids, as well as lanosterol, lupeol, and urs-12-en-28-al [16].

These findings indicate that *P. terebinthus* L. galls constitute a valuable reservoir of bioactive compounds, underscoring their potential in food safety, agricultural applications, and pharmaceutical development.

## 3. Applications in Functional Foods and Nutraceuticals

Various technological approaches have been employed to enhance the stability, functionality, and bioactivity of *P. terebinthus* L. extracts and derivatives across food and nutraceutical applications (Table 2). These methods aim to improve bioactive compound retention, extend shelf life, and optimize functional properties for diverse formulations. Despite its extensive traditional use and demonstrated bioactive potential, *P. terebinthus* L. remains underutilized in modern food and nutraceutical applications. Limited commercial awareness, coupled with a lack of standardized production methods and scalability challenges, has hindered its broader adoption. Increasing industry engagement, consumer education, and investment in sustainable sourcing strategies could significantly enhance its recognition and utilization in functional formulations.

The following sections provide a detailed overview of key applications, emphasizing the significance of encapsulation, food packaging, fermentation, and other innovative techniques in maximizing the potential of *P. terebinthus* L. in the food and health industries. The following sections provide a detailed overview of key applications, emphasizing the significance of encapsulation, food packaging, fermentation, and other innovative techniques in maximizing the potential of *P. terebinthus* L. in the food and health industries.

### 3.1. Encapsulation for Aroma and Nutrient Retention

Encapsulation has proven to be an effective strategy for preserving aroma compounds and enhancing storage stability in *P. terebinthus* L. formulations. An oil-in-water emulsion stabilized with a gum arabic-to-maltodextrin ratio of 75:25 demonstrated high stability with minimal creaming. Among the tested encapsulation techniques, spray-drying with an ultrasonic nozzle achieved the highest encapsulation efficiency (>90%) and maximum aroma retention (73.19%), significantly outperforming freeze-drying (24.45%) and spray-freeze-drying (14.23%). Notably, α-pinene and linalool exhibited nearly 100% retention, underscoring the potential of optimized encapsulation for protecting volatile bioactive compounds [6].

Further research has demonstrated that drying conditions play a critical role in encapsulation efficiency and product stability. The use of inulin at 170 °C resulted in optimal encapsulation efficiency and superior color retention, whereas maltodextrin was the least effective carrier. Additionally, color stability was influenced by drying temperature, with gum arabic enhancing red–green values, while inulin and maltodextrin led to a decline in this parameter [51]. These results suggest that careful optimization of encapsulation conditions can significantly enhance the stability, bioavailability, and functional properties of *P. terebinthus* L. oil, reinforcing its potential as a valuable ingredient in food and nutraceutical applications.

In addition to encapsulation, *P. terebinthus* L. extracts have been successfully integrated into food packaging materials, demonstrating potential as active components in biopolymer-based films. A bilayer film containing 15% fruit extract exhibited strong mechanical properties, excellent water resistance, and a high polyphenol content, making it particularly suitable for protecting oxidation-sensitive foods. However, further research is required to standardize these extracts and ensure regulatory compliance for broader food applications [43].

Apart from its applications in food preservation and packaging, *P. terebinthus* L. has demonstrated potential in enhancing the functional properties of food emulsions. When incorporated into meat-based emulsions at concentrations ranging from 0% to 0.5%, it significantly improved emulsion capacity (234 mL oil/g protein) and stability (75%), with the most pronounced effects observed at 0.3% inclusion. Notably, roasted terebinth exhibited superior emulsion stability, viscosity, and pseudo-plasticity compared to its unroasted counterpart, suggesting its potential as a natural stabilizer for high-fat emulsified foods [11].

These findings suggest that *P. terebinthus* L. extracts can be effectively integrated into food formulations to enhance both stability and functionality, with promising applications in encapsulation, packaging, and emulsified food systems. However, further research is required to optimize processing conditions and establish standardized protocols to facilitate their commercial implementation in the food industry.

### 3.2. Use of P. terebinthus L. Resin as an Immobilization Support for Lactobacillus casei in Dairy Products

The resin of *P. terebinthus* L. has been extensively investigated as a natural matrix for probiotic immobilization, demonstrating its ability to enhance bacterial survival, improve food safety, and influence sensory attributes in dairy applications. Initial studies in yogurt production revealed that resin-assisted immobilization significantly improved probiotic viability (>7 log CFU/g after 60 days at 4 °C) while simultaneously inhibiting the growth of pathogenic microorganisms, including *Salmonella*, *Staphylococci*, and coliforms. Additionally, the resin contributed to the aromatic composition of yogurt, with α-pinene and β-pinene imparting a distinctive flavor profile, reinforcing its potential for functional dairy formulations [13].

Further research expanded its application to myzithra cheese, where its impact was assessed across four variations: (i) cheese with resin-encapsulated *L. casei*, (ii) free *L. casei* with resin, (iii) free *L. casei* without resin, and (iv) traditional myzithra. The resin exhibited strong antimicrobial properties, effectively suppressing yeast and fungal growth while maintaining probiotic viability above 9 log CFU/g. Additionally, encapsulation further enhanced bacterial retention, and resin-containing cheeses developed a distinct mastic gum aroma and smooth texture, reinforcing their commercial potential as premium functional dairy products [44].

More recently, *P. terebinthus* L. resin has been incorporated into functional whey beverages, where immobilized *L. casei* remained highly viable (>1 × 10^6^ CFU/g) for 30 days at 4 °C, with no detectable spoilage microorganisms. The presence of terpenes likely contributed to both its antimicrobial activity and aromatic enhancement. These results highlight the potential of resin-based probiotic beverages within the framework of sustainable dairy waste valorization and circular economy principles [3].

### 3.3. Applications in Fermentation and Alcoholic Beverages

The resin of *P. terebinthus* L. has been utilized as a natural immobilization matrix for *Saccharomyces cerevisiae* AXAZ-1 cells in successive fermentation processes. When applied to sugar-based fermentation media, the resin-immobilized yeast facilitated efficient fermentation within 54 h, across a temperature range of 14–28 °C. The resulting fermented beverages remained stable for 90 days without spoilage, likely due to the high concentration of terpenoids and phenolic compounds. These findings highlight the potential of *P. terebinthus* L. resin as a biocatalyst, particularly for the production of low-alcohol beverages with enhanced aromatic properties, making it commercially viable in the expanding low-alcohol beverage market [14].

Further studies evaluated the use of *P. terebinthus* L. resin in 27 consecutive fermentation cycles of white must at varying temperatures (28, 21, 14, and 7 °C), where the immobilized yeast exhibited high operational stability. Compared to free yeast cells, resin-immobilized *S. cerevisiae* demonstrated shorter fermentation times and increased ethanol productivity, thereby improving the overall efficiency of the process. Additionally, terpenoids and polyphenols extracted from the resin were detected in the resulting wines, contributing to their preservation for up to 35 days at room temperature and 95 days at 4 °C, eliminating the need for potassium metabisulfite as a preservative. These bioactive compounds not only extended wine stability but also imparted a unique and enhanced sensory profile, reinforcing the potential of *P. terebinthus* L. resin in natural wine preservation and aroma enhancement [15].

### 3.4. Applications in Food Preservation and Antimicrobial Activity

The biological evaluation of silver nanoparticles (AgNPs) synthesized using *P. terebinthus* L. extract has demonstrated notable antimicrobial, antioxidant, and anticancer properties. These biogenic AgNPs exhibited strong antibacterial activity against both Gram-positive and Gram-negative bacteria, highlighting their potential as natural antimicrobial agents. Additionally, they displayed potent antioxidant capacity, as evidenced by their DPPH radical scavenging activity, and exhibited high anticancer activity against the MCF-7 breast cancer cell line at a concentration of 25 µg/mL. These findings suggest that AgNPs synthesized from *P. terebinthus* L. extract could serve as promising medicinal compounds with applications in food preservation, pharmaceuticals, and biomedical research [45].

### 3.5. Functional Foods and Antioxidants

*Pistacia terebinthus* L. resin has been identified as a nutrient-rich additive with potential applications in functional food fortification. Studies have demonstrated its effectiveness in noodle enrichment, where formulations incorporating raw and roasted terebinth (100 °C) at a 10% inclusion level resulted in reduced phytic acid content while significantly increasing ash, protein, fat, total dietary fiber, total phenolic content, and antioxidant capacity (*p* < 0.05). Sensory evaluation further confirmed its positive impact on consumer acceptance, reinforcing its potential as a functional food ingredient [46]. These findings suggest that *P. terebinthus* L. resin could serve as a valuable enhancer of antioxidant activity and dietary fiber content in staple food products.

Additionally, *P. terebinthus* L. has been successfully incorporated into ice cream as a natural antioxidant and flavor enhancer. Notably, seeds roasted at 125 °C exhibited the highest antioxidant activity. Following milling, shelling, and incorporation prior to pasteurization, the ingredient demonstrated strong potential as a functional additive in frozen desserts, contributing both nutritional and sensory benefits [47].

### 3.6. Applications in Animal Nutrition and Health

The potential of *P. terebinthus* L. as a functional feed additive has been explored in aquaculture and poultry nutrition, demonstrating positive effects on growth performance, metabolism, and immune function.

A 63-day feeding trial conducted on juvenile rainbow trout (*Oncorhynchus mykiss*) supplemented with *P. terebinthus* L. fruit extract at 0.1%, 0.5%, and 1% inclusion levels resulted in significant improvements in weight gain, feed conversion efficiency, and digestive enzyme activity (pepsin, lipase, trypsin, and amylase). While the hematological parameters remained unchanged, the activity of antioxidant enzymes (superoxide dismutase, glutathione peroxidase, glucose-6-phosphate dehydrogenase) increased, and lipid peroxidation in liver and muscle decreased, suggesting oxidative stress protection. Additionally, immune markers (nitroblue tetrazolium reduction, myeloperoxidase, lysozyme) were enhanced, further reinforcing the potential of *P. terebinthus* L. extract as a feed additive to improve fish health in aquaculture [48].

In poultry nutrition, dietary supplementation of 30 g/kg *P. terebinthus* L. seed meal in laying hens (Babcock breed) improved egg quality, particularly freshness (Haugh unit) and yolk color, while eggshell strength and weight remained unaffected. These benefits were most pronounced after 20–30 days of storage, suggesting potential applications in the poultry industry [49]. Furthermore, feeding hens diets containing 0–5% *P. terebinthus* L. seed meal over eight weeks resulted in significantly higher egg production at 3% and 4% inclusion levels, while egg weight increased with 2% or more. Importantly, no adverse effects were observed on blood parameters (glucose, cholesterol, etc.) or oxidative stress markers, indicating that *P. terebinthus* L. supplementation can enhance egg production and quality without negatively affecting hen metabolism [50].

These findings highlight the nutritional and functional benefits of *P. terebinthus* L. in animal feed formulations, supporting its potential role in aquaculture and poultry production as a natural growth promoter and antioxidant-rich additive. However, to fully harness the bioactive potential of this species and facilitate its integration into diverse commercial applications, several important research gaps remain.

## 4. Future Research Directions

Despite the promising applications of *P. terebinthus* L., several research gaps must be addressed to support its broader adoption in the food, nutraceutical, and pharmaceutical industries:

- Optimization of processing techniques: while current studies have explored encapsulation, food packaging, and probiotic immobilization, further research is needed to refine processing parameters—such as carrier materials, drying conditions, and extraction methods—to maximize bioactive compound retention and functional efficacy.
- Standardization and quality control: the phytochemical profile of *P. terebinthus* L. varies widely depending on geographical origin, plant part, and harvest stage. Establishing standardized protocols for chemical profiling, authentication, and quality control is essential to ensure consistency, reproducibility, and efficacy in commercial applications.
- Yield and extraction efficiency: given that *P. terebinthus* L. is not yet commercially cultivated, future studies should focus on evaluating the yield variability and extraction efficiency of essential oils and bioactive compounds, particularly from fruits and leaves. This will be crucial for determining the plant’s economic feasibility and scalability for industrial use.
- Long-term stability and shelf-life studies: investigations into the stability of bioactive constituents in different storage conditions and food matrices are needed to validate the long-term effectiveness of *P. terebinthus* L. in functional food and preservation systems.
- Regulatory and safety assessments: although initial studies support the safety and health benefits of *P. terebinthus* L. extracts, comprehensive toxicological evaluations and regulatory assessments are required to establish safe usage levels and gain approval for food and nutraceutical applications.
- Allergenicity and oxidative stability of volatile compounds: given that major volatile constituents of *P. terebinthus* L. essential oils—such as limonene, α-pinene, and β-pinene—are known to oxidize into allergenic compounds, future research should assess their stability under storage and processing conditions relevant to food systems. Although these substances are approved as flavorings, evaluating their allergenic potential, degradation behavior, and safe inclusion levels is critical for ensuring consumer safety, particularly in functional food formulations and edible packaging applications.
- Mechanistic insights into bioactivity: more in-depth pharmacological and biochemical research is necessary to clarify the specific mechanisms through which *P. terebinthus* L. compounds exert their antimicrobial, antioxidant, and other health-promoting properties.
- Sustainability and commercial viability: as a wild-growing species, *P. terebinthus* L. presents challenges related to sustainable harvesting and supply chain development. Future efforts should investigate environmentally responsible collection practices and cost-effective, scalable extraction technologies to support commercialization.
- Expansion into new applications: additional studies are encouraged to explore novel delivery systems and product formats, including incorporation into functional beverages, dietary supplements, edible films, and intelligent food packaging materials.

By addressing these priorities, *P. terebinthus* L. may be successfully developed into a sustainable, multifunctional resource with broad utility across the food, nutraceutical, and pharmaceutical sectors.

## 5. Conclusions

*Pistacia terebinthus* L. is a promising, yet underutilized, source of bioactive compounds, with demonstrated potential in functional foods, nutraceuticals, and food preservation. Its diverse plant parts—fruits, seeds, resin, leaves, and galls—exhibit unique biochemical profiles that contribute to antioxidant, antimicrobial, and other health-promoting properties. Recent technological advances, including encapsulation, edible packaging, and probiotic immobilization, have enhanced the applicability of its extracts in modern food systems. Despite these promising findings, several challenges remain. The lack of commercial cultivation, variability in chemical composition, and limited safety assessments restrict its widespread adoption. Future efforts should focus on optimizing extraction methods, ensuring regulatory compliance, and implementing sustainable harvesting practices to support long-term use. Addressing these gaps will enable the transformation of *P. terebinthus* L. from a traditional medicinal plant into a commercially viable, multifunctional ingredient for food and health industries.

## Figures and Tables

**Table 1:** Bioactive compounds and functional potential of *P. terebinthus* L. across different plant parts.

Plant Part	Ingredient/Method	Main Compounds	Functional Potential	References
Fruits	Cold-pressed oil (traditional aqueous-assisted cold pressing)	Crude fat (39% *), crude protein (10%), crude fiber (11%); fatty acids: oleic acid (47%), linoleic acid (23%), palmitic (22%); vitamins: vitamin C (>1%), γ-tocopherol (>1%); minerals: potassium (<1%), calcium (>1%), sodium (>1%), phosphorus (>1%)	Alternative supplement, industrial oil, antioxidant, supports bone health, electrolyte balance, nerve and muscle function, metabolic activity	[4,5,23,24]
	Fatty acids (solvent extraction with *n*-hexane)	Oleic acid (53%), palmitic acid (21%), linoleic acid (19%)	Cognitive and cardiovascular health, neuroprotective effects, energy source	[25]
	Essential oil (hydrodistillation)	(*Z*)-β-Ocimene (2–45%), D-limonene (16–24%), α-pinene (13–37%), *p*-cymen-8-ol (11%), *p*-anisaldehyde (7%)	Antimicrobial agent food preservative, aroma enhancer	[5,26,27]
	Phenolic compounds (sonication with methanol)	Rutin (1%), quercetin (1%), luteolin (<1%), gallic acid (<1%), syringic acid (<1%), catechol (<1%)	Antioxidant, antimicrobial, antidiabetic, flavor enhancer, food preservation	[5,24,28]
Seeds	Cold-pressed oil (Soxhlet method)	Crude oil (47%), crude protein (9%), carbohydrates (21%); fatty acids: oleic acid (45%), palmitic acid (24%), linoleic acid (24%)	Edible oil, functional food applications	[29,30]
	Protein-rich oil (protein extraction from cold-pressed oil)	Protein (54–66%), carbohydrate (23–34%)	Emulsifying and foaming properties, food industry	[12]
Resin	Triterpenoids (fractionated to acidic and neutral fractions)	Isomasticadienonic acid (26% *w*/*w* **), 28-norolean-17-en-3-one (18%), masticadienonic acid (7%)	Anti-inflammatory, antimicrobial, anti-tumor, antioxidant, and gastroprotective activities	[31]
Leaves	Essential oil (hydrodistillation)	α-Pinene (33–47%), limonene (17–29%), β-pinene (3–12%)	Fungicidal activity, food preservative	[26,32]
	Essential oil (hydrodistillation)	α-Pinene (35%), camphene (2%), β-pinene (5%), terpinolene (35%), β-phellandrene (5%)	Fungicidal activity	[32]
	Triterpenoids (solvent extraction with ethyl acetate)	Masticadienediol (n.d. ***), isomasticadienonic acid (n.d.), masticadienonic acid (n.d.), masticadienolic acid (n.d.), tirucallone (n.d.), oleanolic acid (n.d.), ursolic acid (n.d.), morolic acid (n.d.)	Anti-inflammatory, nutraceutical applications; targeting food allergy management and immune health	[8]
	Flavonoids (aqueous Reflux Extraction)	Kaempferol (4%) and rutin (2%)	Diabetes and obesity management, colorectal cancer prevention	[33]
	Phenolics (maceration with methanol)	Gallic, digallic, protocatechuic, and *p*-coumaric acids (n.d), procyanidin B (n.d.), and various flavonoids (n.d.) such as taxifolin, myricetin, quercitrin, quercetin, cosmosiin, and luteolin	Antioxidant, neuroprotective, glucose metabolism regulation, food preservation	[34]
Galls	Essential oil (hydrodistillation)	α-Pinene (53–66%), limonene (17–23%), β-pinene (0–11%)	Natural preservative	[26]
	Fatty acids/triterpenoids (Soxhlet extraction with ethanol)	Lauric acid (n.d.), myristic acid (n.d.), lanosterol (n.d.), lupeol (n.d.)	Natural preservative	[16]
	Phenolics (Soxhlet extraction with methanol)	Gallic acid (n.d.), rutin (n.d.), caffeoylquinic acid (n.d.)	Potential chemopreventive agent	[17]

* Concentrations are given as % on a dry matter basis; ** % *w*/*w* refers to weight/weight percentage within the isolated terpenoid fraction; *** n.d. stands for not determined concentration.

**Table 2:** Technologies applied to *P. terebinthus* L. plant parts in food and nutraceutical applications.

Technology	Plant Part	Key Findings
Encapsulation	Fruit oil	Spray-drying with ultrasonic nozzle achieved >90% encapsulation efficiency; α-pinene and linalool retained nearly 100% [6]
Food packaging	Fruit extracts	Bilayer film with 15% fruit extract showed strong mechanical properties and high water resistance [43]
Emulsion stabilization	Fruits and seeds	Enhanced emulsion capacity and stability in meat emulsions; roasted terebinth provided better results [11]
Probiotic immobilization in dairy products	Resin	Improved probiotic viability (>7 log CFU/g in yogurt), antimicrobial effects in cheese, and enhanced aroma [3,13,44]
Fermentation carrier for yeast	Resin	Maintained yeast viability for 90 days, enhanced aroma compounds, and improved fermentation efficiency [14,15]
Food preservation	Extract (silver nanoparticles)	Strong antimicrobial and antioxidant activity; effective against Gram-positive and Gram-negative bacteria [45]
Functional food fortification (noodles, ice cream)	Resin and seeds	Increased antioxidant activity, dietary fiber, and protein content; improved sensory properties [46,47]
Animal nutrition (fish feed, poultry feed)	Fruits and seeds	Improved growth, digestion, and immune response in fish; increased egg production and weight in poultry [48,49,50]

## Data Availability

No new data were created or analyzed in this study. Data sharing is not applicable to this article.

## References

[1] Chaves Lobón N., González Félix M., Alías Gallego J.C. (2023). Comparison of the Allelopathic Potential of Non-Native and Native Species of Mediterranean Ecosystems. Plants.

[2] Bozorgi M., Memariani Z., Mobli M., Salehi Surmaghi M.H., Shams-Ardekani M.R., Rahimi R. (2013). Five *Pistacia* Species (*P. vera*, *P. atlantica*, *P. terebinthus*, *P. khinjuk*, and *P. lentiscus*): A Review of Their Traditional Uses, Phytochemistry, and Pharmacology. Sci. World J..

[3] Schoina V., Terpou A., Papadaki A., Bosnea L., Kopsahelis N., Kanellaki M. (2020). Enhanced Aromatic Profile and Functionality of Cheese Whey Beverages by Incorporation of Probiotic Cells Immobilized on *Pistacia terebinthus* Resin. Foods.

[4] Badem A. (2021). Some Traditional Terebinth Dishes in Turkey and Their Health Effects. J. Gastron. Hosp. Travel.

[5] Fidan M.S., Baltacı C., Öz M., Akar Z. (2023). Chemical Composition of *Pistacia terebinthus* L. and Its Phytochemical and Biological Properties. BioResources.

[6] Yaman D.M., Koçak Yanık D., Elik Demir A., Uzun Karka H., Güçlü G., Selli S., Kelebek H., Göğüş F. (2023). Effect of Encapsulation Techniques on Aroma Retention of *Pistacia terebinthus* L. Fruit Oil: Spray Drying, Spray Freeze Drying, and Freeze Drying. Foods.

[7] Batovska D., Inbar M. (2024). Beyond the Nut: *Pistacia* Leaves as Natural Food Preservatives. Foods.

[8] Rauf A., Patel S., Uddin G., Siddiqui B.S., Ahmad B., Muhammad N., Mabkhot Y.N., Hadda T.B. (2017). Phytochemical, Ethnomedicinal Uses and Pharmacological Profile of Genus *Pistacia*. Biomed. Pharmacother..

[9] Bagheri F., Hassanshahi G., Khanamani Falahatipour S., Dini A., Mohamadi M., Ahmadi Z., Noroozi M., Khanamani Falahatipour S. (2021). Effects of Pistachios and Their Different Plant Parts on Various Disorders: Evidence about Their Therapeutic Effects on Diabetes Mellitus, Gastrointestinal and Liver Disorders, as well as Blood Pressure. Pistachio Health J..

[10] Kamiloglu S., Ozdal T., Bakir S., Capanoglu E. (2022). Bioaccessibility of Terebinth (*Pistacia terebinthus* L.) Coffee Polyphenols: Influence of Milk, Sugar and Sweetener Addition. Food Chem..

[11] Alagöz E., Sarıçoban C. (2024). The Effects of Adding Ground Terebinth Fruits on the Emulsification, Microstructural, and Flow Properties of Meat Emulsions. Food Sci. Biotechnol..

[12] Ozgolet M., Cakmak Z.H.T., Bozkurt F., Sagdic O., Karasu S. (2024). Response Surface Optimization of Protein Extraction from Cold-Pressed Terebinth (*Pistacia terebinthus* L.) Oil Byproducts: Physicochemical and Functional Characteristics. J. Food Sci..

[13] Schoina V., Terpou A., Angelika-Ioanna G., Koutinas A., Kanellaki M., Bosnea L. (2015). Use of *Pistacia terebinthus* Resin as Immobilization Support for *Lactobacillus casei* Cells and Application in Selected Dairy Products. J. Food Sci. Technol..

[14] Kallis M., Sideris K., Kopsahelis N., Bosnea L., Kourkoutas Y., Terpou A., Kanellaki M. (2019). *Pistacia terebinthus* Resin as Yeast Immobilization Support for Alcoholic Fermentation. Foods.

[15] Kallis M., Boura K., Karabagias I.K., Kanellaki M., Koutinas A.A. (2022). Beneficial Effects of *Pistacia terebinthus* Resin on Wine Making. Appl. Sci..

[16] Akpulat S., Tiras M., Sahinkaya M.S., Akpulat H.A. (2021). Antimicrobial Effect of *Pistacia terebinthus* (Turpentine Tree) Galls and GC/MS Analysis. Turk. J. Biodivers..

[17] Nazım B., Ismaıl B., Houari T., Zakaria N. (2020). In Vitro Antimitotic Activity of Gall Extract of *Pistacia terebinthus*. Curr. Perspect. Med. Aromat. Plants.

[18] Mandalari G., Barreca D., Gervasi T., Roussell M.A., Klein B., Feeney M.J., Carughi A. (2022). Pistachio Nuts (Pistacia vera L.): Production, Nutrients, Bioactives and Novel Health Effects. Plants.

[19] Soulaidopoulos S., Tsiogka A., Chrysohoou C., Lazarou E., Aznaouridis K., Doundoulakis I., Tyrovola D., Tousoulis D., Tsioufis K., Vlachopoulos C. (2022). Overview of Chios Mastic Gum (*Pistacia lentiscus*) Effects on Human Health. Nutrients.

[20] Ben Ahmed Z., Yousfi M., Viaene J., Dejaegher B., Demeyer K., Vander Heyden Y. (2021). Four *Pistacia atlantica* Subspecies (*atlantica*, *cabulica*, *kurdica* and *mutica*): A Review of Their Botany, Ethnobotany, Phytochemistry and Pharmacology. J. Ethnopharmacol..

[21] Benmahieddine A., Belyagoubi-Benhammou N., Belyagoubi L., Amari N.O., Zerey-Belaskri A.E., Gismondi A., Di Marco G., Canini A., Habi S., Atik Bekkara F. (2023). Leaf-Buds of *Pistacia atlantica*: A Novel Source of Bioactive Molecules with High Anti-Inflammatory, Antioxidant, Anti-Tyrosinase, and Antimicrobial Properties. Physiol. Mol. Biol. Plants.

[22] Belyagoubi-Benhammou N., Belyagoubi L., Benmahieddine A., El Zerey-Belaskri A., Di Marco G., D’Agostino A., Canini A., Gismondi A. (2024). Nutraceutical Content and Biological Properties of Lipophilic and Hydrophilic Fractions of the Phytocomplex from *Pistacia atlantica* Desf. Buds, Roots, and Fruits. Plants.

[23] Tufan T., Arslan C., Daş A. (2017). Effects of Terebinth (*Pistacia terebinthus* L.) Fruit Oil Supplementation to Diets on Fattening Performance, Carcass Characteristics, Blood Parameters, and Breast Meat Fatty Acid Composition in Japanese Quails (*Coturnix coturnix japonica*). Kafkas Universitesi Vet. Fakultesi Derg..

[24] Özcan M.M., Al Juhaimi F., Uslu N., Ahmed I.A.M., Babiker E.E., Osman M.A., Gassem M.A., Alqah H.A.S., Ghafoor K. (2020). Effect of Sonication Process of Terebinth (*Pistacia terebinthus* L.) Fruits on Antioxidant Activity, Phenolic Compounds, Fatty Acids, and Tocopherol Contents. J. Food Sci. Technol..

[25] Hacıbekiroğlu I., Yılmaz P.K., Haşimi N., Kılınç E., Tolan V., Kolak U. (2015). In Vitro Biological Activities and Fatty Acid Profiles of *Pistacia terebinthus* Fruits and *Pistacia khinjuk* Seeds. Nat. Prod. Res..

[26] Pulaj B., Mustafa B., Nelson K., Quave C.L., Hajdari A. (2016). Chemical Composition and In Vitro Antibacterial Activity of *Pistacia terebinthus* Essential Oils Derived from Wild Populations in Kosovo. BMC Complement. Altern. Med..

[27] İnan M. (2021). Seasonal Variation of Fatty and Essential Oil in Terebinth (*Pistacia terebinthus* L.) Fruit. Not. Bot. Horti Agrobot. Cluj-Napoca.

[28] Akyuz M., Yabo-Dambagi L., Kilic T., Cakir A. (2022). Antidiabetic, Neuroprotective, and Antioxidant Potentials of Different Parts of *Pistacia terebinthus* Fruits. S. Afr. J. Bot..

[29] Kaya F., Özer A. (2015). Characterization of Extracted Oil from Seeds of Terebinth (*Pistacia terebinthus* L.) Growing Wild in Turkey. Turk. J. Sci. Technol..

[30] Kaya F., Özer A. (2020). A Study on the Investigation of Diffusion Coefficient for Oil Extraction from Terebinth (*Pistacia terebinthus* L.) Seeds. Curr. Phys. Chem..

[31] Assimopoulou A.N., Papageorgiou V.P. (2005). GC-MS Analysis of Penta- and Tetra-Cyclic Triterpenes from Resins of *Pistacia* Species. Part II. Pistacia terebinthus var. Chia. Biomed. Chromatogr..

[32] Piras A., Marzouki H., Maxia A., Marengo A., Porcedda S., Falconieri D., Salgueiro L. (2017). Chemical Characterization and Biological Activity of Leaf Essential Oils Obtained from *Pistacia terebinthus* Growing Wild in Tunisia and Sardinia Island. Nat. Prod. Res..

[33] Hamlat N., Alqaraleh M., Kasabri V., Mizher H., Hassani A., Afifi F., Al Alawi S., Ouafi S., Khwaldeh A. (2024). Dual Amylase/Glucosidase Inhibition, Antilipolytic and Antiproliferative Potential of the Aerial Parts of *Pistacia atlantica*, *Pistacia lentiscus*, and *Pistacia terebinthus* on an Obesity-Related Colorectal Cancer Cell Line Panel. Curr. Issues Pharm. Med. Sci..

[34] Uysal S., Sinan K.I., Jekő J., Cziáky Z., Zengin G. (2022). Chemical Characterization, Comprehensive Antioxidant Capacity, and Enzyme Inhibitory Potential of Leaves from *Pistacia terebinthus* L. (Anacardiaceae). Food Biosci..

[35] Aşan Özüsağlam M. (2024). Investigation of New Potential Uses of Menengiç (*Pistacia terebinthus*) for Various Areas. Kahramanmaraş Sütçü İmam Üniv. Tarım Doğa Derg..

[36] Barbouchi M., Elamrani K., El Idrissi M., Choukrad M. (2019). The Effect of Solvent Extracts on the Measurement of Total Polyphenol, Flavonoid, and Tannin Contents and Its Relation to Antioxidant Capacities from Various Parts of Terebinth (*Pistacia terebinthus* L.). Mor. J. Chem..

[37] Aqil M., Ahad A., Sultana Y., Ali A. (2007). Status of Terpenes as Skin Penetration Enhancers. Drug Discov. Today.

[38] Gómez-Favela M.A., Santos-Ballardo D.U., Bergés-Tiznado M.E., Ambriz-Pérez D.L., Heredia J.B., Gutiérrez-Grijalva E.P., Licea-Claverie A., Gutierrez-Uribe J.A., Patra J.K. (2023). Nanoformulations Applied to the Delivery of Terpenes. Nanotechnology in Biomedicine, Phytochemical Nanodelivery Systems as Potential Biopharmaceuticals.

[39] Karlberg A.-T., Lepoittevin J.-P. (2021). One Hundred Years of Allergic Contact Dermatitis Due to Oxidized Terpenes: What We Can Learn from Old Research on Turpentine Allergy. Contact Dermatitis.

[40] Sukakul T., Bruze M., Mowitz M., Antelmi A., Bergendorff O., Björk J., Dahlin J., Hamnerius N., Hauksson I., Isaksson M. (2022). Contact Allergy to Oxidized Linalool and Oxidized Limonene: Patch Testing in Consecutive Patients with Dermatitis. Contact Dermat..

[41] Hu W., Meng X., Wu Y., Li X., Chen H. (2024). Terpenoids, a Rising Star in Bioactive Constituents for Alleviating Food Allergy: A Review about the Potential Mechanism, Preparation, and Application. J. Agric. Food Chem..

[42] Nazım B., Houssem F., Ismaıl B., Yassıne M. (2021). Antimicrobial Activity of Leaf, Fruit, and Gall Extract of *Pistacia terebinthus* Growing in Tessala. Curr. Perspect. Med. Aromat. Plants.

[43] Dodange S., Shekarchizadeh H., Kadivar M. (2023). Development and Characterization of Antioxidant Bilayer Film Based on Poly Lactic Acid–Bitter Vetch (*Vicia ervilia*) Seed Protein Incorporated with *Pistacia terebinthus* Extract for Active Food Packaging. Curr. Res. Food Sci..

[44] Schoina V., Terpou A., Bosnea L., Kanellaki M., Nigam P.S. (2018). Entrapment of *Lactobacillus casei* ATCC393 in the Viscus Matrix of *Pistacia terebinthus* Resin for Functional Myzithra Cheese Manufacture. LWT.

[45] Naghmachi M., Raissi A., Baziyar P., Homayoonfar F., Amirmahani F., Danaei M. (2022). Green Synthesis of Silver Nanoparticles (AgNPs) by *Pistacia terebinthus* Extract: Comprehensive Evaluation of Antimicrobial, Antioxidant and Anticancer Effects. Biochem. Biophys. Res. Commun..

[46] Köten M., Ünsal A.S. (2021). Nutritional, Chemical, and Cooking Properties of Noodles Enriched with Terebinth (*Pistacia terebinthus*) Fruits Roasted at Different Temperatures. Food Sci. Technol..

[47] Ergenekon M., Akbulut Çakır Ç. (2024). Impact of *Pistacia terebinthus* on the Antioxidant Activity and Total Phenolics of Ice Cream Depending on Roasting Conditions and Incorporation Time. Eur. J. Food Sci. Technol..

[48] Ali G.M.A., Bilen S., Güney K. (2022). Growth Promoter, Immunostimulant and Antioxidant for Rainbow Trout (*Oncorhynchus mykiss*): Terebinth (*Pistacia terebinthus*) Extract. Mar. Sci. Technol. Bull..

[49] Gültepe E.E., Çetingül I.S., Uyarlar C., Iqbal A., Rahman A., Hacisalihoglu S., Ozcinar U., Bayram I. (2018). Effects of *Pistacia terebinthus* Seed Meal and Different Storage Times on Egg Quality of Laying Hens. R. Bras. Zootec..

[50] Çetingül I.S., Gültepe E.E., Rahman A., Iqbal A., Uyarlar C., Hacisalihoglu S., Özcinar Ü., Bayram I. (2020). *Pistacia terebinthus* as a Dietary Supplement for Laying Hens. S. Afr. J. Anim. Sci..

[51] Poyrazoglu E., Ozat E., Coksari G., Ozat E., Konar N. (2017). Effect of Various Process Conditions on Efficiency and Colour Properties of *Pistacia terebinthus* Oil Encapsulated by Spray Drying. ETP Int. J. Food Eng..

